# Risk factors associated with injection initiation among drug users in Northern Thailand

**DOI:** 10.1186/1477-7517-3-10

**Published:** 2006-03-14

**Authors:** Yingkai Cheng, Susan G Sherman, Namtip Srirat, Tasanai Vongchak, Surinda Kawichai, Jaroon Jittiwutikarn, Vinai Suriyanon, Myat Htoo Razak, Teerada Sripaipan, David D Celentano

**Affiliations:** 1 Johns Hopkins University, Bloomberg School of Public Health, Baltimore, MD, USA; 2 Research Institute for Health Sciences, Chiang Mai University, Chiang Mai, Thailand; 3 Ministry of Public Health, Nonthaburi, Thailand; Family Health International, Bangkok, Thailand

## Abstract

**Background:**

Circumstances surrounding injection initiation have not been well addressed in many developing country contexts. This study aimed to identify demographic factors, sexual behaviors and drug use characteristics related to injection initiation among drug users in northern Thailand.

**Methods:**

A cross-sectional survey was conducted among 2,231 drug users admitted to the Northern Drug Treatment Center in Mae Rim, Chiang Mai, Thailand, between February 1, 1999 and December 31, 2000. A multiple logistic regression was employed to identify the independent effects from potential risk factors of transition into injection.

**Results:**

After controlling for other covariates, being 20 years of age or older, single, ever receiving education, urban residence, and having a history of smoking or incarceration were significantly associated with higher likelihood of injection initiation. Multiple sex partners and an experience of sex abuse were associated with an increased risk of injection initiation. Comparing to those whose first drug was opium, individuals using heroin as their initiation drug had greater risk of injection initiation; conversely, those taking amphetamine as their first drug had less risk of injection initiation. Age of drug initiation was negatively associated with the risk of injection initiation: the older the age of drug initiation, the less the risk of injection initiation.

**Conclusion:**

Injection initiation was related to several demographic factors, sexual behaviors and drug use characteristics. Understanding these factors will benefit the design of approaches to successfully prevent or delay transition into injection.

## Background

A range of studies have examined the factors associated with transition from non-injection into injection drug use; however most of them are conducted in the developed world [1-5]. This transition is a critical point in a drug user's career for many reasons, particularly because it is characterized by a heightened risk of acquiring HIV and other blood-borne pathogens [6-8]. The process of initiating injection is complicated and informed by a myriad of individual, social-culture, and environmental factors. Demographic characteristics significantly associated with transition into injection in developed countries have included: being of younger age [5,9]; being female [10]; ethnic minority [3,11]; housing instability [5]; and being a high school dropout [3]. Having a current steady sexual relationship with a partner who is an IDU has long been observed as a facilitating factor for transition into drug injection [12]. Sexual abuse alone has put drug users at a higher likelihood of injection initiation [5,13]. Many studies have recorded the independent effect of drug use characteristics on onset of injection, such as types of recently used drugs [5,12]; intensiveness and/or length of drug use [9-12,14,15]; and current treatment status [1]. At the social-culture level, low socio-economic status has consistently been found to be one of the most important factors in initiating injection in the developed world [2,4].

An exploration of the circumstances around the critical time of initiating injection practices in developing countries as related to medical outcomes has not been well addressed. Limited evidence from China [16,17] indicated that risk factors of injection initiation in developing countries could be as similar as those in developed countries like younger age, ethnic minority and short duration of heroin use. However, Lai et al [17] reported that being male related to higher risk of injection initiation, which has not been shown in other studies [10,18]. The situation in Thailand is of particular interest because injecting drug use continues to be a leading risk factor for HIV in the country that was previously characterized as having a heterosexual epidemic [19]. After implementing the 100% condom use program in the 1990s, by 2002 the national HIV prevalence was about 2% and prevalence among 21 year-old military conscripts was under 1%, having dropped from 4% in the mid-1990s [20]. Unfortunately, similar prevention efforts have not been waged to reduce HIV infection among injection drug users (IDUs), partly due to lack of knowledge about injection initiation.

Drug use, especially use of methamphetamines, has increased rapidly in recently years in Thailand, particularly among young people [21-23]. Sattah and colleagues [23] reported among adolescent students in northern Thailand, 41.3% males and 19.0% females ever used methamphetamines. The risk of HIV infection is high among drug users in Thailand. The reported HIV incidence was about 3%~19% [22,24,25]. As shown in other places, IDUs had a much higher risk of HIV infection than non-IDUs in northern Thailand [24-26]. Srirak et al. [21] reported that among female drug users in northern Thailand the HIV prevalence among IDUs was six more times than that among non-IDUs (25% vs. 4.1%). While it is feasible to deliver harm reduction programs in Thailand [27], they are currently very limited [26,28,29]

The purpose of this study was to identify demographic and drug use factors related to onset of injection among drug users, who were admitted to one large drug treatment center, in order to provide information to develop harm reduction strategies with the hope of preventing and controlling drug injection as well as HIV infection.

## Methods

### Study population

This study was a part of a cohort study, the Opiate Users Research (OUR) Study, which was initiated at the Northern Drug Treatment Center (NDTC), located in Mae Rim, Chiang Mai Province, Thailand in 1999. NDTC is the largest drug treatment center in northern Thailand. Between February 1, 1999 and January 31, 2000 2,845 individuals were admitted for inpatient treatment, which consisted of 21-day detoxification. OUR Study inclusion criteria were: being 12 years or older; being admitted for opiate or methamphetamine dependence; agreeing to receive pre- and post-test HIV counseling and venipuncture for antibody testing for HIV infection; providing informed consent for those 18 and older and parental consent and assent for individuals below the age of 18; and completion of a behavioral questionnaire. Of 2,149 persons determined to be eligible, 1,865 (87%) enrolled in this study. An additional 366 IDUs admitted to NDTC between February and December 2000 were also enrolled in the study. A total of 2,231 drug users were therefore included into this analysis. The study was approved by the institutional review boards of the Royal Thai Ministry of Public Health, Research Institute of Health Sciences, Chiang Mai University and the Johns Hopkins University.

### Data collection

The study questionnaire was developed based on information from pilot studies (in-depth interviews and focus groups) and a systematic literature review. A formal pretest was conducted in a similar setting. The questionnaire has been translated into local languages and back-translated into English without information loss. Interviewers received formal training and were fluent in 12 local languages. Study participants completed the interview in their preferred language.

After explaining study requirements and obtaining informed consent, an interviewer conducted a 30–40 minute risk factor questionnaire in a private setting. Following HIV pre-test counseling, a serum specimen was collected; one week later patients were given their HIV test results during HIV post-test counseling. The questionnaire ascertained information on demographics, tobacco and alcohol use, non-parenteral modes of HIV transmission, and a comprehensive drug use and sexual behavior history. Drug use history included lifetime use of marijuana, opium, heroin, methamphetamines, inhalants (paint thinner and glue), sedatives and hypnotics, and other drugs. Age at first use, priority of reasons for first use, and use in the past year and in the past three months were assessed for each drug. Route of drug administration (inhalation, ingestion, and injection), frequency of use, amount taken per dose, and purchase price of each drug were assessed for all drugs used in the past 3 months. Injection history was also obtained, including characteristics of first injection, syringe sharing history, and syringe disinfection practices. Drug treatment and family and friends' drug use were also examined.

### Dependent and independent variables

Independent variables included demographic factors, sexual behaviors and drug use characteristics, specifically age, gender, ethnicity, education, employment, residence, number of sex partner, sex abuse history, types of drugs ever used, and age of drug initiation. The dependent variable was initiation of injection.

### Statistical methods

Univariate analysis was conducted to describe the study sample's demographics, sex and drug use behaviors. Continuous variables (age, number of lifetime sex partners and age of drug initiation) were roughly categorized based on their quartiles in the statistical analyses. Logistic regression was employed to assess both unadjusted and independent effects of potential risk factors. Variables that were included in the initial and final multiple logistic models were based on statistical significance (*p *< 0.05) and/or scientific importance. Interaction terms were systematically tested. A manual back-and-forth process was employed to obtain the final parsimonious model. SAS software (version 9.1, SAS Institute, Cary, North Carolina) was used to conduct the analysis.

## Results

### Demographic factors, sexual behaviors and drug use characteristics

Descriptive results are located in Table 1. The median age of this sample was 29.3 years with an inter-quartile range (IQR) of 21.4~38.5 years. Nearly 90% of participants were male and more than half (55.5%) were Thai, with the remaining being ethnic minorities. In this analysis, the marital statue of single (58.5%) included those who were single, separated, divorced or widowed. Two-thirds of the sample lived in rural areas. About one-third had no formal education and only 16.6% completed 12 or more years of education. Most of the participants were employed (81.8%). Both smoking (93.0%) and drinking (80.9%) were very common. About one-third of participants ever gave a history of incarceration. The HIV prevalence of this sample was 15.6%.

**Table 1:** Demographic factors, sexual behaviors and drug use characteristics of 2,231 drug users admitted to NDTC, Chiang Mai, Thailand

	Number (%) (n = 2231)
Age (years), Median (IQR)	29.3 (21.4~38.5)
Male	2005 (89.9)
Thai ethnicity	1238 (55.5)
Single*	1301 (58.3)
Rural residence	1685 (75.5)
Education	
No schooling	725 (32.5)
≤ 6 years	500 (22.4)
7~11 years	635 (28.5)
12 or More years	371 (16.6)
Employed	1824 (81.8)
Ever smoking	2074 (93.0)
Ever drinking	1805 (80.9)
Ever been in jail/prison	732 (32.8)
HIV positive	347 (15.6)
Number of sex partners, median (IQR)	5.0 (1.0~10.0)
Ever selling sex	40 (1.8)
Ever sex abused	31 (1.4)
The first drug ever used	
Opium	755 (33.8)
Heroin	326 (14.6)
Amphetamine	478 (21.4)
Marijuana	541 (24.3)
Others**	131 (5.9)
Age of drug initiation (years), median (IQR)	17.3 (15.1~22.7)
Ever injection	879 (39.4)

* Single includes single, separated, divorced, and widow

** Others include inhalants and tranquilizers/sedatives

Half of the participants had five or more lifetime sex partners with an IQR of 1~10. Forty participants (1.8%) ever sold sex for money or drug and thirty one (1.4%) reported they were sexually abused.

Opium (33.8%) was the most common initiation drug, followed by marijuana (24.3%), amphetamine (21.4%) and heroin (14.6%). The median age of drug initiation was 17.3 years with an IQR of 15.1~22.7 years. In total, 879 participants (39.4%) ever injected drugs.

### Correlates of injection initiation

Table 2 shows results from bivariate analysis. The unadjusted odds ratio [UOR] indicated that older age (20 years of age or over), male, Thai ethnicity and single civil status were associated with a higher risk of injection initiation. Drug users living in rural areas had less likelihood to initiate injection than those living in urban areas (UOR = 0.39). Comparing to those with no formal schooling, any formal education increased the likelihood of injection initiation. A history of smoking, drinking or incarceration related to higher risk of injection initiation. Multiple sex partners and a history of selling sex or reporting sex abuse were associated with increased likelihood of injection initiation. Comparing to opium users, using marijuana, heroin or other drugs had a higher risk of injection initiation while using amphetamine as the initiation drug was associated with a much lower risk. Comparing to drug users at younger age (less than 16 years old), those at 16 to 18 years of age had a higher risk of injection initiation, while those at 24 years of age or older had a lower risk.

**Table 2:** Unadjusted odds ratio for factors associated with initiation of injection

	Unadjusted odds ratio (95% CI)
Age	
Under 20 years old	Reference
20~29 years old	8.3 (6.0~11.4)
30~39 years old	6.6 (4.8~9.2)
40 years old or older	3.2 (2.3~4.5)
Male	2.2 (1.6~3.1)
Thai ethnicity	1.4 (1.2~1.7)
Single	1.6 (1.3~1.9)
Rural residence	0.39 (0.32~0.47)
Education	
No schooling	Reference
≤ 6 years	2.5 (1.9~3.1)
7~11 years	1.4 (1.1~1.8)
12 or more years	2.5 (1.9~3.2)
Employed	0.96 (0.77~1.2)
Ever smoking	7.3 (4.2~12.7)
Ever drinking	2.1 (1.6~2.6)
Ever in jail	7.2 (5.9~8.7)
No. of lifetime sex partners	
One or no partner	Reference
2~5 partners	2.4 (1.9~3.1)
6~10 partners	3.9 (2.9~5.2)
11 or more partners	6.1 (4.7~7.9)
Ever selling sex	2.9 (1.5~5.6)
Ever sex abused	2.8 (1.4~6.0)
Type of initiation drug	
Opium	Reference
Marijuana	3.4 (2.7~4.2)
Heroin	3.4 (2.6~4.5)
Amphetamine	0.13 (0.085~0.20)
Others	3.9 (2.6~5.7)
Age of drug initiation	
Under 16 years old	Reference
16~18 years old	1.5 (1.2~1.8)
19~23 years old	1.0 (0.80~1.3)
24 years old or older	0.39 (0.30~0.50)

Table 3 contains results from multiple logistic models examining demographic, sexual, and drug use characteristics associated with injection initiation. In the presence of other variables, the demographics that were found significantly associated with initiation of injection were: being 20 years or older (Adjusted Odds Ratio [AOR] = 4.3 for those 20~29 years old; AOR = 6.0 for those 30~39 years old; and AOR = 5.3 for those 40 years of age or older); being single, (AOR = 1.7); and being urban (change the direction of the AOR). Those who had more education (compared to those without schooling, AOR = 1.7 for those with 6 or less years of education; AOR = 1.8 for those with 7~11 years of eduction; and AOR = 12 or more years of education), ever smoking (AOR = 2.9) or being incarcerated (AOR = 5.0) all had an increased risk of starting injection.

**Table 3:** Adjusted odds ratio for factors associated with initiation of injection

	Adjusted odds ratio (95% CI)
Age	
Under 20 years old	Reference
20~29 years old	4.3 (2.7~6.8)
30~39 years old	6.0 (3.6~10.2)
40 years old or older	5.3 (3.0~9.3)
Male	
Thai ethnicity	
Single	1.7 (1.3~2.2)
Rural residence	0.50 (0.37~0.66)
Education	
No schooling	Reference
≤ 6 years	1.7 (1.2~2.3)
7~11 years	1.8 (1.2~2.7)
12 or more years	1.5 (1.0~2.3)
Employed	
Ever smoking	2.9(1.5~5.3)
Ever drinking	
Ever in jail	5.0 (4.0~6.4)
No. of lifetime sex partners	
One or no partner	Reference
2~5 partners	1.1 (0.83~1.6)
6~10 partners	1.5 (1.0~2.2)
11 or more partners	1.9 (1.4~2.7)
Ever selling sex	
Ever sex abused	3.3 (1.2~9.1)
Type of initiation drug	
Opium	Reference
Marijuana	0.98 (0.68~1.4)
Heroin	2.7 (1.9~3.8)
Amphetamine	0.098 (0.057~0.17)
Others	1.2 (0.70~2.1)
Age of drug initiation	
Under 16 years old	Reference
16~18 years old	0.83 (0.60~1.1)
19~23 years old	0.56 (0.39~0.79)
24 years old or older	0.25 (0.16~0.37)

In terms of sex behaviors, more sex partners implied higher risk of transition into injection (comparing to those who did not have any or only one sex partner, AOR = 1.5 for those with 6~10 partners; and AOR = 1.9 for those with 11 or more partners). Having a history of sex abuse was significantly associated with heightened risk of starting injection (AOR = 3.3).

In terms of drug use, the type of initiation drug mattered: comparing to opium users, heroin use was related to higher likelihood of transition into injection (AOR = 2.7), while amphetamine use was associated with a lower risk of injection initiation (AOR = 0.098). Age of drug initiation had a significant impact on the likelihood of starting injection: compared to those who began drug use at less than 16 years, the risk of initiating drug use at the age of 19~23 years old was about half (AOR = 0.56) as likely and about one-quarter as likely (AOR = 0.25) for those initiating drug use at the age of 24 years or older.

## Discussion

The study's findings indicate that demographic factors, sexual behaviors and drug use characteristics account for some of the risks in transition into injection. Drug users who were older or single, obtained more education, lived in urban areas, ever smoked or had an experience of incarceration were more likely to initiate injection. Multiple sex partners or a history of sex abuse related to a higher likelihood of starting injection; using amphetamines instead of heroin as the starting substance of abuse and later drug initiation were associated with a lower risk of injection initiation.

Contrary to a study conducted among street youths (14–25 years of age) in Montreal, Canada [5], our data showed that older age was associated with a higher risk of initiation into injection. The obvious difference in study settings and age structure between the two studies may account for some of the difference in the age effects. Our study participants were from a resource-limited country and older than those in the Canada study. Furthermore, all our participants were drug users, whose age was apparently associated with both drug use history and mode of drug administration. Old drug users might have involved in the drug culture for an extended period of time. Thus, it is not surprising that older drug users had great opportunity to transition into injection. In this analysis the effect of drug use history was partly controlled by including age of drug initiation into the final model; however, the impact of initial and sequential drug use modes on transition into injection was not extensively explored.

As a contextual factor, neighborhood (residence) has been shown as an importance determinant of starting injection [30]. Since most studies have been conducted in developed countries, the effect of rural residence on initiation of injection is unknown. Our data demonstrated that people living in rural areas had half the risk to initiate injection as those in urban areas after controlling for other confounders such as education. Ethnicity and employment were initially included into the model but were dropped due to statistical non-significance. In current analysis, postal code was employed to define the types of residence, and current residence was used as a surrogate for the neighborhood that influenced the occurrence of injection, which may have been inaccurate. If more detailed information is available, it would be helpful to explore how contextual factors influence and shape drug users' behavior and injection practices in Thailand. As Galea et al. [31] point out, to fully understand drug use behaviors, researchers should address not only individual risk behaviors but also contextual factors such as social norms and neighborhood socioeconomic status by means of statistical methods like multilevel analysis.

Not reported from some studies [5,32], we found multiple sex partners (6 or more) to be a risk factor of transition into injection. However, in line with other studies [5,13], the experience of sexual abuse increased the likelihood of injection initiation. Though not explored in this study, others [33,34] have demonstrated that negative life events and risky behaviors following sex abuse could be possible mechanisms in the casual chain. In our study we cannot determine the temporal relationships between sex behaviors and injection initiation, which makes it hard to establish the causal pathway.

Comparing to opium, taking heroin as the initiation drug was associated with a larger likelihood of transition into injection, while starting with amphetamines lowered the risk. Mode of drug administration and multiple drug use following initiation drug might account for their specific effect. In the past three months, 72.9% of heroin users injected the drug, while only 7.5% of amphetamine users were injectors (data not shown). Furthermore, those starting with amphetamines tended to stick with it; only 12.3% of them ever used heroin. Though behaviors in the past three months were not necessarily constant over time, they at least indicated some drug use pattern in our sample. Among individuals starting from marijuana or others (inhalants and tranquilizers/sedatives), 59.7% and 54.2% ever tried opium sometime late respectively, which might partly explain the non-significant difference in risk of injection between marijuana or others with opium. Multiple drug use and the intensity of drug use were not examined in the current analysis, though many other studies [10,11,14] have indicted that they are related to a higher risk of initiating injection.

In our study, younger age of drug initiation was significantly associated with a higher risk of injection initiation even after controlling for the effect of age and other demographics, which has not been reported in other studies [5,32]. The interaction between age and age of drug initiation was examined and the effect was not statistically significant. As indicated by our results, those starting drug use at less than 16 years had four times the risk of transition into injection than those starting at the age 24 years or older. One implication for this finding is to focus health education programs on youths.

The HIV prevalence at study enrollment among drug users in our sample was 15.6%, which was comparable to the results from other studies in similar settings [22,24,25]. Compared to the estimated HIV prevalence of 1~2% among the adult population in Thailand [35], the risk of HIV infection among drug users was clearly quite high. Further analyses (data not shown) indicated that the risk of HIV infection varied by age, age at drug initiation, injection status and types of recently used drugs. Drug users 20 years old or over had a higher prevalence of HIV infection than those younger than 20 years old (18.4% vs. 3.7%, P < 0.0001), while those starting drugs at younger age (under 16 years old) had a higher risk of HIV infection than those starting drug use at older ages (age of 16 years old or over) (17.9% vs. 7.3%, P < 0.0001). Considering the correlations between age, length of drug use (age of drug initiation), and the accumulated risk of HIV infection during the entire course of drug use, the observed differences in the risk of HIV infection between different age groups and length of drug use were reasonable but unacceptable. HIV prevention programs and health education projects need to be enhanced and should target the population at risk, especially youth. The HIV prevalence among IDUs was 35.2% in our sample, which was 12.6 times higher than that of non-IDUs (2.8%) (P < 0.0001). In terms of drug use in the past 3 months, HIV prevalence was highest among users of other drugs (inhalants and tranquilizers/sedatives, 35.1%), followed by heroin users (29.9%), amphetamine users (16.0%), marijuana users (15.0%) and opium users (10.4%). A study from the same region [21] reported the HIV prevalence was 25% among heroin injectors and 4.1% among opium or methamphetamine smokers. Gender could be one reason to the observed difference in HIV risk since the sample for the above study only included females. However, in our sample 44.2% of participants were multi-drug users. In the past 3 months, 61.7 % of inhalants and tranquilizers/sedatives users, 46.4% of marijuana users, 40.8% of amphetamine users, and 21.9% opium users also reported using heroin, which matched well with the observed HIV risk for individual drugs. As shown in elsewhere [36], multiple drug use was associated with a higher risk of HIV infection, especially when heroin injection was involved. This finding indicates the importance of addressing the issue of multiple drug use in harm reduction programs, and not just focusing on injection of heroin or methamphetamines.

The interpretation of these results is subject to certain limitations. One concern is the representativeness of the sample. Recruited from one large drug treatment center, drug users in our study could be a highly selected group with relatively high risks. Data have shown that a history of drug treatment may reflect past severity of drug dependence, itself a predictor of transition into injection [12,14]. Secondly, all behavioral data were from self-reports that might be inaccurate. Nevertheless, all interviewers were asked to evaluate the quality of the interview at the end of each interview and most reported good cooperation and judged the data to be moderately to very reliable. A study by McElrath and colleagues [37] also showed that retrospective self-reports from injection drug users had good reliability and consistency. The extent of under-reporting of socially unacceptable activities or over-reporting was unknown and inaccessible. Thirdly, some informative variables such as types, modes, and intensiveness of drug use prior to injection initiation were not assessed. Exploring these risk factors could shed additional understanding of the process of initiating injection, and might also benefit the design of appropriate harm reduction program to target the individuals at risk. Finally, the cross-sectional design of this study limited our ability to determine causality; some potential risk factors for transition into injection might result from the fact that the participant initiated injection and then identified those experiences a risk context, such as being in jail. Furthermore, the use of a cross-sectional design could induce recall bias and survival bias.

Despite these limitations, we have identified several correlates of injection initiation among drug users in this Southeast Asian context. Understanding the factors related to initiation into injection may be an important tool in guiding how we modify current harm reduction strategies or design new approaches to more specifically target high-risk populations and high-risk behaviors to delay or prevent the initiation of injection.

## Conclusion

Injection initiation was related to several demographic factors, sexual behaviors and drug use characteristics in northern Thailand. The information from this study could be employed to improve current efforts in harm reduction in order to better serve the people at risk.

## Competing interests

The author(s) declare that they have no competing interests.

## Authors' contributions

DDC, VS and JJ conceived of the study and directed study operations; NS, TV and MHR were instrumental in the conduct of the field operations, and participated in interpretation of the data. YC, SGS, SK and TS conducted the statistical analysis. DDC drafted the manuscript; all authors read and approved the final manuscript.
